# Supplementary material for: Polish medical students facing the pandemic—Assessment of resilience, well-being and burnout in the COVID-19 era
Source: PLoS One. 2022 Jan 24;17(1):e0261652. doi: 10.1371/journal.pone.0261652 (PMC8786167; doi:10.1371/journal.pone.0261652)
Supplement: S1 File — (DOCX) [file pone.0261652.s001.docx]

**Assessment of burnout, well-being and mental resilience among medical students in the era of the COVID-19 pandemic**

Dear Colleagues,

we are students of the Medical University of Lodz. As part of our research activities at the Student Research Club at the Department of Nephrology, Hypertensiology and Kidney Transplantology, we deal with the issues of burnout, well-being and mental resilience of medical students in the era of the SARS-CoV-2 coronavirus pandemic.

Although there are many reports on the impact of the current situation on burnout symptoms and other aspects of mental well-being in healthcare professionals, little is known about the situation of medical students. This fact prompted us to conduct this study.

The survey is completely anonymous. The data collected in this project will be used for research purposes only. The study protocol received a positive opinion of the Ethics Committee at the Medical University of Lodz (consent number: RNN / 275/20 / KE).

Solving the questionnaire takes approx. 20 minutes.

We cordially invite you to complete the survey.

Thank you for your attention and your time

Authors

**Do you give your consent to participate in the study?**

Yes

No, I am leaving the survey.

**Gender:**

Female

Male

Other

**Age:**

18-20 y.o.

21-23 y.o.

24-26 y.o.

More than 26 y.o.

**Year of studies:**

I

III

IV

V

VI

**Universities:**

Uniwersytet Medyczny w Białymstoku

Uniwersytet Mikołaja Kopernika w Toruniu - Collegium Medicum w Bydgoszczy

Gdański Uniwersytet Medyczny

Śląski Uniwersytet Medyczny w Katowicach

Śląski Uniwersytet Medyczny, Wydział Nauk Medycznych w Zabrzu

Collegium Medicum Uniwersytetu Jagiellońskiego w Krakowie

Uniwersytet Medyczny w Lublinie

Uniwersytet Medyczny w Łodzi

Uniwersytet Medyczny im. Karola Marcinkowskiego w Poznaniu

Warszawski Uniwersytet Medyczny

Uniwersytet Medyczny im. Piastów Śląskich we Wrocławiu

Uniwersytet Warmińsko-Mazurski w Olsztynie

Uniwersytet Jana Kochanowskiego w Kielcach

Uniwersytet Rzeszowski

Uniwersytet Zielonogórski w Zielonej Górze

Krakowska Akademia im. Andrzeja Frycza Modrzewskiego

Uniwersytet Opolski

Uniwersytet Technologiczno-Humanistyczny im. Kazimierza Pułaskiego w Radomiu

Wyższa Szkoła Techniczna w Katowicach

Uniwersytet im. Kardynała Stefana Wyszyńskiego w Warszawie

Uczelnia Medyczna im. Marii Skłodowskiej-Curie w Warszawie

Pomorski Uniwersytet Medyczny

Uczelnia Łazarskiego w Warszawie

**I MBI -GS(S)**

**II MSWBI**

**III RS-14**

**IV SELF-CREATED PART OF THE SURVEY**

**Psychological help/medication/stimulants**

1. Have you ever sought psychological help either from psychologist or psychiatrist?

Yes

No

1. Have you ever taken medication prescribed by a psychiatrist?

Yes

No

1. During your studies, did you take any over-the-counter sedative medication (tablets, herbs, etc.)?

Yes

No

1. Have you noticed higher consumption of medication mentioned in the previous question during current or previous academic year?

Yes

No

1. Are you diagnosed with a mental disorder?

Yes, I do have a depressive disorder

Yes, I do have an anxiety disorder

Yes, I do have a psychotic disorder

Yes, I do have a personality disorder

Yes, I do have a stress disorder

Other

No

1. If so, did your condition deteriorate during COVID-19 pandemic due to the disorder you are diagnosed with?

Yes

No

1. How did COVID-19 pandemic affect your self-esteem?

Negative

Positive

No effect

1. Do you drink alcohol, smoke cigarettes or take other stimulants more often during COVID-19 pandemic?

Yes

No

1. Has the pandemic and the changes related to it (limitation of interpersonal relationship, government restrictions, fear of illness, etc.) caused you to reach for any form of psychological help (psychological consultation, medical consultation, helpline or other)?

Yes

No

**Volunteering / work / classes**

1. Did you have any practical classes in the 2020/2021 academic year (e.g. laboratory classes, training on phantoms, practical anatomy classes)?

No

Yes and the numer of classes was in accordance with course sylabus

Yes, but the numer of classes was limited

1. Did you have any clinical classes (classes in hospital wards in the presence of patients) in the academic year 2020/2021?

No

Yes and the numer of classes was in accordance with course sylabus

Yes, but the numer of classes was limited

1. Have you worked as a volunteer in a health care unit dealing with patients suffering from COVID-19?

Yes

No

1. Are you currently working as a volunteer in a health care unit dealing with COVID-19 patients?

Yes

No

1. Are you going to volunteer at a health care unit dealing with COVID-19 patients?

Yes

No

1. What is your attitude towards being referred to work in a health care unit dealing with patients suffering from COVID-19?

I am glad that I can help.

I think it is my duty.

I will only do this to avoid the consequences.

I am willing to quit medical studies to avoid it.

1. Are you concerned about being referred to work for a COVID-19 health unit?

Yes

No

1. If so, what are your biggest concerns about volunteering for the COVID-19 health care unit?

No personal protective equipment;

The possibility of becoming infected with the virus and transmitting it to loved ones;

Spending time to volunteer instead of studying for tests / exams;

I am afraid of the potential consequences related to criminal liability for inadequate assistance to patients;

I have no concerns.

1. Do you think that your current level of theoretical and practical knowledge is sufficient to work taking care of people suffering from COVID-19?

Yes

No

1. Have you been able to do your summer internship this year?

Yes, I did it at the designated ward;

Yes, but they were modified (triage, administrative assistance, etc.);

I haven't been able to do any internship this year.

**SARS-CoV-2 infection**

1. Have you been infected with the SARS-CoV-2 virus?

Yes

No

1. If so, was the infection associated with any medical conditions?

The infection was asymptomatic;

The course of the disease was mild;

The course of the disease was severe;

I was not infected.

1. Has anyone in your family / close friends been infected with SARS-CoV-2?

Yes

No

1. Has anyone in your family / close friends been suffering from COVID-19?

Yes

No

1. Has anyone in your family / close friends died because of COVID-19?

Yes

No

**E-learning**

1. What form of e-learning suits you best?

Recorded lectures / seminars;

Shared presentations;

Live classes via on-line messaging;

Tasks for self-completion.

1. What form of e-learning do you most often participate in?

Recorded lectures / seminars;

Shared presentations;

Live classes via on-line messaging;

Tasks for self-completion.

1. Do you think that online classes may prevent you from acquiring the necessary practical skills during your studies?

Yes

No

1. Do you think that online classes may have a negative impact on the amount of necessary theoretical knowledge you will acquire during your studies?

Yes

No

1. Are you afraid that by limiting the numer of clinical classes you will get worse mark at LEK (Final Medical Examination)?

Yes

No

1. In the era of online classes, are you more likely to procrastinate / postpone doing something?

Yes

No

1. Do you think that your motivation to study has decreased by conducting online classes?

Yes

No

1. Do you study less since you started remote learning than you did before?

I study more than before I started learning online;

I study less than before I started learning online;

I study comparable amount of time.

1. Does limiting direct contact with other students as a result of limiting the numer of stationary classes increase your feeling of loneliness?

From 1 to 5, where 1 is „Not at all” and 5 i „A lot”.

1

2

3

4

5

**Feelings / Opinion on the medical profession**

1. Do you think that social aversion and distrust towards doctors increased during the pandemic?

Yes

No

1. If so, does this affect your enthusiasm for entering this profession in the future?

Yes

No

1. Have the pandemic and challenges being faced by the health care system affected your enthusiasm for your future profession?

Yes, it affected positively;

Yes, it affected negatively;

No, it didn't, I've been burned out for a long time;

No, it didn't, I'm still just as interested in working as a doctor.

1. Has the pandemic situation in the Polish health care system make you consider working abroad in the future?

I had planned to work abroad before;

I am not considering working abroad;

I am starting to consider working abroad;

I don't think about it.

1. Are you considering leaving university and not finishing your studies, due to the pandemic?

Yes

No
